# Discrepancy of social cognition between bipolar disorders and major depressive disorders

**DOI:** 10.1002/brb3.3365

**Published:** 2023-12-31

**Authors:** Yun‐Hsuan Chang, Chu‐Ling Yu, Chih‐Chun Huang, Tzu‐Yun Wang, Isabel Dziobek, Hsien‐Yuan Lane

**Affiliations:** ^1^ Institute of Gerontology, College of Medicine National Cheng Kung University Tainan Taiwan; ^2^ Institute of Behavioral Medicine, College of Medicine National Cheng Kung University Tainan Taiwan; ^3^ Department of Psychology National Cheng Kung University Tainan Taiwan; ^4^ Institute of Genomics and Bioinformatics College of Life Sciences, National Chung Hsing University Taichung Taiwan; ^5^ Department of Psychology, College of Medical and Health Sciences Asia University Taichung Taiwan; ^6^ Department of Psychiatry National Cheng Kung University Hospital, Dou‐Liou Branch Yunlin Taiwan; ^7^ Department of Psychiatry, National Cheng Kung University Hospital, College of Medicine National Cheng Kung University Tainan Taiwan; ^8^ Berlin School of Mind and Brain Humboldt‐Universität zu Berlin Berlin Germany; ^9^ Graduate Institute of Biomedical Sciences China Medical University Taichung Taiwan; ^10^ Department of Psychiatry and Brain Disease Research Center China Medical University Hospital Taichung Taiwan; ^11^ Department of Psychiatry China Medical University Hsinchu Hospital Hsinchu Taiwan

**Keywords:** bipolar disorder, depression, mentalizing, movie for social cognition (MASC), social cognition

## Abstract

**Background:**

The research landscape examining social cognition (SC) impairment in patients with major depressive disorders (MDD) and bipolar disorders (BD) is notably scarce. Presently, assessments predominantly rely on static stimuli and self‐reported measures, which may not capture the dynamic dimensions of social cognition.

**Objectives:**

This study aimed to validate the Chinese version of Movie Assessment of Social Cognition (MASC‐CH) and to investigate whether MDD and BD exhibit distinct patterns of SC impairments, shedding light on potential differences between these two mood disorders.

**Methods:**

The study encompassed 197 participants, aged 18–65, distributed as follows: 21 BD, 20 MDD, and 156 healthy controls (HC). We focused on examining “cognitive” and “emotional” SC scores and “undermentalizing” and “overmentalizing” error patterns, with nonsocial inference as a control. Additional assessments included the Reading Mind in the Eyes Test (RMET) and the Mayer–Salovey–Caruso Emotional Intelligence Test (MSCEIT). We also explored the association between depression severity (measured by the Hamilton Depressive Rating Scale, HDRS) and distinct SC dimensions between MDD and BD.

**Results:**

The MASC‐CH exhibited strong validity and reliability for SC assessment. In group comparisons, BD participants scored significantly lower on MASC‐CH, while the MDD group scores were not significantly different from HC. Specifically, BD individuals had notably lower cognitive SC scores and made more undermentalizing and absence of mentalizing errors than MDD and HC. Additionally, a negative correlation between HDRS score and overmentalizing was observed in BD, not in the MDD.

**Conclusions:**

The findings indicate that depression severity scores in BD were inversely related to MASC‐CH scores. In contrast, this relationship was not observed in the MDD group. These results underscore the importance of SC impairments as distinguishing characteristics of both BD and MDD. It provides valuable insights into the distinct social‐cognitive profiles of both mood disorders.

## Graphical abstract

1. Patients with bipolar disorders exhibit impairment of social cognition 2. Patients with bipolar disorders displayed more errors with undermentalizing compared to patients with major depressive disorders

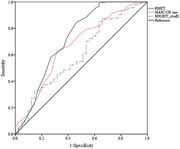

## 1 INTRODUCTION

Mood disorders, impacting various aspects of life, particularly social interactions, have recently been scrutinized concerning social cognition in patients. Social cognition involves comprehending and responding to others' thoughts and feelings, crucial for social interactions. Mood disorders, such as major depressive disorders (MDD) and bipolar disorders (BD), are marked by disturbances across various domains, encompassing interpersonal and social functioning. They are often typified by challenges in adapting to social and occupational roles (Kessler et al., 2006; Romera et al., 2010; Wells et al., 1989).

Social cognition (SC) has been defined as the ability to identify and understand mental states, including cognitive and emotional characteristics from which we infer both our own and other mental states to predict behaviors or reactions (Fiske & Taylor, 2013). The five theoretical approaches of social cognition include the theory of mind (ToM), emotional intelligence (EI), social perception, social knowledge, and attribution bias (Green et al., 2008); whether these five areas intersect or are distinct has been a subject of much debate (Samamé, 2013). The ToM refers to our ability to identify thoughts, intentions, and emotions as belonging either to ourselves or to someone else, and this has been defined as the core construct in SC (Premack & Woodruff, 1978). A two‐dimensional model of SC was suggested and examined that the low‐level processing involves perception and attention in processing emotional stimuli. In contrast, the high‐level processing involves mentalizing and strategy along with affective processing (Etchepare & Prouteau, 2017).

In recent years, SC impairment in mental disorders (e.g., schizophrenia) has been a focus in SC research since these patients have poor social interactions and significant cognitive dysfunction. Studies exploring theory of mind (ToM) in remitted patients with MDD and bipolar depression present diverse outcomes. While one study identified deficits in complex ToM tasks requiring simultaneous perspective integration (second‐order false‐belief questions), it found no impairment in simpler ToM tests involving single‐character perspective inference (first‐order false‐belief questions) (Inoue et al., 2004). Nevertheless, deficits become apparent in both first‐order and second‐order theory of mind (ToM) questions, with the impairment being more pronounced in the cognitively demanding second‐order ToM tasks (McKinnon et al., 2010). In contrast, Kerr et al. (2003) found deficits in both first‐order and second‐order ToM tests among actively depressed or manic bipolar disorder (BD) patients, while remitted patients exhibited no impairment.

A review study reported 29 articles (i.e., 12 studies on ToM, 11 on emotion recognition, and 6 on social judgment) with a meta‐analysis showing evidence that individuals with BD have significant deficits, including emotion recognition, theory of mind, and social judgment, and most prominent during mood episodes, even persist during periods of remission (Gillissie et al., 2022). Studies on emotional intelligence, patients with BD have been revealed their EI impairmentat their remitted status regardless of BDI or BDII (Liu et al., 2021), especially the impairment in strategic EI area. In addition, the EI deficit was found as mood‐dependent, instead of subtype dependent in patients with BD (Kuo et al., 2021). Patients with MDD often interpret social‐cognitive cues differently from healthy individuals, showing a mood‐congruent bias and difficulty with complex mental state interpretation. Social‐cognitive performance inversely correlates with depression severity, persisting even in remission (Weightman et al., 2014). With deficits in higher‐level function of SC, such as managing emotions in BD could be permanent (Samamé, 2013, 2015), and persistent in remitted BD (Liu et al., 2021; Montag et al., 2010). However, the low‐level SC function, such as the perception and identification of emotions, may not be affected by the mood phases in BD (Kuo et al., 2021; Liu et al., 2021) and in MDD (Żuchowicz et al., 2018).

Previous studies have found two dimensions of SC in different brain areas. Affective SC was related to an increased activation in the medial frontal cortex and temporoparietal junction (Amodio & Frith, 2006; Gallagher & Frith, 2003). MDD showed an increased activation in the postcentral gyrus, as compared to HC participants, on attention and emotion recognition tasks (Cerullo et al., 2014), while the BD patients exhibited abnormalities in the anterior temporal cortical thickness, prefrontal cortical, orbital cortex, and limbic system on a set of tasks related to emotional processing (McIntosh et al., 2008; Quidé et al., 2020; Strakowski et al., 2005). It may be that MDD and BD use different brain networks to process emotions, so that their SC performance may be theoretically and psychopathologically different.

Neuroimaging studies have shown that individuals with mood disorders exhibit heightened activation in emotion‐related brain regions and reduced activity in the frontal areas linked to emotion regulation and higher cognitive functions during social cognition tasks. This indicates a lack of higher‐order cognitive control over emotional regions during social cognition in these patients. However, this neural pattern can be influenced by factors like illness severity, comorbid conditions, medications, and cognitive load (Cusi et al., 2012).

Both BD and MDD patients suffer from depression; in BD, depression severity has been correlated with strategic EI area impairment while age and mania/hypomania severity has been related with experiential EI area impairment (Kuo et al., 2021). MDD has also been reported to have an association between depression and EI. Depression severity appears to play different roles in MDD versus BD. The influence of depression on social‐cognitive capabilities is not thoroughly elucidated. Nevertheless, some evidence hints at the presence of a milder form of social cognition impairment in individuals with MDD. While MDD is predominantly recognized by emotional symptoms like persistent low mood and anhedonia, individuals experiencing depression also exhibit substantial and pervasive challenges in their interpersonal interactions. Patients with mood disorders and its different subtypes may have different domains of mentalization disability and have difficulties in SC dimensions (van Neerven et al., 2021).

According to Achim et al. (2013), the definition of mentalization is the capacity to comprehend and interpret the mental states, beliefs, desires, and intentions of oneself and others. It allows individuals to ascribe mental content to behaviors, fostering empathy and social cognition (Achim et al., 2013). The Movie Assessment of Social Cognition (MASC) was originally developed with these definitions and considers dynamic and relative sources when a person is engaged in mentalizing judgments using ecologically valid stimulus material (Dziobek et al., 2006; Wacker et al., 2017).

The original MASC has been widely translated into several languages, including English, Italian, and Spanish, and has been applied in Western cultures. Several studies have used the MASC to evaluate social cognition performance, specifically in people with autism spectrum disorder, schizophrenia, and borderline personality disorder (Dziobek et al., 2006; Fossati et al., 2018; Lahera et al., 2014). To date, studies of social cognition in patients with MDD and BD are scant, and whether mentalization performance could be used to distinguish between MDD and BD is unclear (Malle, 2021). Thus far, the MASC has not been tested for validity and reliability in Eastern cultures or in patients with different types of mood disorders, particularly those with core symptoms of emotional dysregulation and difficulties in social interactions. To the best of our knowledge, this study was the first to translate the MASC into Chinese and validate its components with other relative social cognition tasks. In addition, the discrepancies in the psychopathology between MDD and BD were investigated in the current study.

## 2 METHODS

### 2.1 Participants

All patients were referred by a senior psychiatrist for an initial diagnosis. Each patient received a structured interview by trained research assistants using the Mini‐International Neuropsychiatric Interview (MINI) (Sheehan et al., 1998) to confirm a diagnosis according to the *Diagnostic and Statistical Manual of Mental Disorders, 5th edition* (DSM‐5). Participants with substance use, neurological, or neurodegenerative disorders were excluded. Both patient groups, their severity of depression was measured using the Hamilton Depression Rating Scale 17 (HDRS) (Hamilton, 1967), and the severity of mania/hypomania in patients with bipolar disorders was evaluated using the Young Mania Rating Scale (YMRS) (Young et al., 1978). According to the previous literature, a YMRS score less than 7 was defined as normal, 8−13 as marginal, 14−20 as mild, 21−26 as moderate, and greater than 38 as severe. Similarly, an HDRS total score of 0−3 was defined as normal, 4−7 as marginal, 8−15 as mild, 16−26 as moderate, and greater than 27 as severe (Furukawa, 2010). Healthy controls were recruited in the community via online advertisements and posters. Those who had other mental disorders, neurological disorders, or first‐degree relatives who had a history of mental disorders were excluded.

### 2.2 Measurements

#### 2.2.1 Movie for the Assessment of Social Cognition (MASC)

The MASC was developed with compositions of a 15‐min short film clip depicting four characters (2 females, 2 males) displaying social interactions (Dziobek et al., 2006), thus comprising more naturalistic stimulus material than other tests that are based on for example photographs, comics, or texts (Dziobek, 2012). The participants were required to analyze the character's thoughts, feelings, and intentions. The MASC was categorized as mental state modalities with ToM variables “cognitive” and “emotional” mental states, and the control condition was demanded as nonsocial inferencing with six questions. Besides the sum score of the 45 items of the MASC with dichotomous choice (“correct”/“inaccurate”) response, the multiple choice version also allowed us to analyze the error patterns to further understanding mentalizing abilities (“undermentalizing” vs. “overmentalizing”) (Montag et al., 2010). The scoring system of MASC contained the following parameters: (1) cognitive and emotional scores, (2) scores by error patterns with undermentalizing, overmentalizing or absence of mental inference strategies, and (3) nonsocial inference as a control factor.

The original reliability of MASC was .84. The verbal transcription and translation of the original German version of the MASC was translated into Chinese by two bilingual interpreters. A panel of experts in clinical mental health and cognitive psychology then analyzed the translation and removed culturally incongruent expressions from the instrument. Finally, based on the methodology from the original study by Dziobek et al. (2006), we analyzed our results against theirs for comparisons of the MASC's psychometric properties and its capacity for discriminating between HC, BD, and MDD.

#### 2.2.2 Mayer–Salovey–Caruso Emotional Intelligence Test (MSCEIT)

The MSCEIT examines several levels of emotional intelligence (EI) including experiential and strategic EI areas (MSCEIT_expEI and MSCEIT_straEI), and their four branches: emotional perception, emotional use, emotional concept, and emotional management (Brackett & Salovey, 2006; Ma et al., 2010; Mayer, 2002; Mayer & Geher, 1996; Mayer & Salovey, 1993; Mayer et al., 1999, 1990, 2008; Mayer et al., 2001, 2003). The traditional Chinese version of MSCEIT was validated and had good reliability according to Cronbach's alpha, .82 (Ma et al., 2010; Mao et al., 2016).

#### 2.2.3 Reading the Mind in the Eyes Test (RMET)

The RMET is composed of 36 photos of eyes, representing different emotional expressions. The participants were required to choose one response according to the description of each photo and to identify the gender represented by the photo as a control score. This task has been suggested to have sensitivity in SC examinations (Baron‐Cohen et al., 1997; Baron‐Cohen et al., 2001; Dziobek et al., 2006).

### 2.3 Ethics

This study was approved by the Institute Review Board (IRB) of University hospital (#NCKUH A‐BR‐108‐001, #NCKUH B‐ER‐108‐448, #CMUH108‐REC3‐024, and #CMUH109‐REC3‐036) and local medical hospitals (#JAH 109‐095).

### 2.4 Statistical analysis

The data were analyzed using the Statistical Program for Social Sciences, version 22.0 (SPSS), and G power analysis for prior power analysis. The statistical significance was set at *p* < .05. Factor analysis and item analysis were first conducted to test the Chinese version of MASC (MASC‐CH). To study the correlation among SC measurements, Pearson's correlation coefficients were calculated. Multivariate analysis of variance (MANOVA) test was conducted to compare the differences among three groups, MDD, BD, and HC. Finally, the receiver operating characteristic (ROC) curve was calculated to evaluate the sensitivity and specificity of MASC‐CH and the comparison of discriminatory powers of the other social cognition measures (RMET and MSCEIT).

For power analysis, the effect size conventions is medium effect size, 0.30 for the χ^2^ test, and for MONOVA between‐subject design with a fixed effect size, 171 participants would have a large effect size = 0.0625 (Buchner et al., 1996). For the unequal numbers of patient group and healthy controls, Scheffee post hoc would be used for multiple comparisons with unequal group numbers.

## 3 RESULTS

### 3.1 Validation and reliability of MASC‐CH

The original MASC was set for a single‐factor assessment, and in the Chinese version, the eigenvalue of the first factor was 5.24 while all other factors had eigenvalue yielded 0.21. Four items showed negative item‐scale correlations, indicating the less contributions to the MASC‐CH score (items, 4, 12, 13, 37). Subsequent analyses excluded these items revealed an improvement in proportion of explained variance. The internal consistency coefficient of MASC‐CH based on Cronbach's alpha was .790, an acceptable reliability. Moreover, the intraclass correlation coefficient for test–retest reliability was .862 (*p* < .0005). Thus, the four items were excluded from the subsequent analyses, and the updated MASC sum score was further referred to the MASC‐CH score. Moreover, positive correlation was found among these SC tasks, MASC‐CH, MSCEIT, and RMET (Table 1).

**TABLE 1:** The correlation matrix among variables (*N* = 156).

	1	2	3	4	5	6	7	8	9	10
1. MASC‐CH sum score	–									
2. MASC‐CH “emotional”	.838^***^	–								
3. MASC‐CH “cognitive”	.955^***^	.639^***^	–							
4. MASC‐CH “overmentalizing”	–.623^***^	–.520^***^	–.597^***^	–						
5. MASC‐CH “undermentalizing”	–.812^***^	–.692^***^	–.770^***^	.230^**^	–					
6. MASC‐CH “absent”	–.711^***^	–.583^***^	–.685^***^	.107	.471^***^	–				
7. MSCEIT total score	.446^***^	.436^***^	.392^***^	–.284^***^	–.319^***^	–.366^***^	–			
8. MSCEIT_ExpEI	.271^**^	.282^***^	.229^**^	–.180^*^	–.189^*^	–.221^**^	.854^***^	–		
9. MSCEIT_straEI	.465^***^	.429^***^	.421^***^	–.265^**^	–.364^***^	–.378^***^	.832^***^	.468^***^	–	
10. RMET	.386^***^	.387^***^	.335^***^	–.235^**^	–.320^**^	–.273^**^	.427^***^	.335^***^	.378^***^	–

* *p* < .05.

** *p* < .001.

*** *p* < .0005.

### 3.2 HC versus patient groups in social cognition

A total of 156 healthy controls (HC) were further compared to the 41 patients with mood disorders. The results showed no significant differences of background information between groups.

**FIGURE 1:**
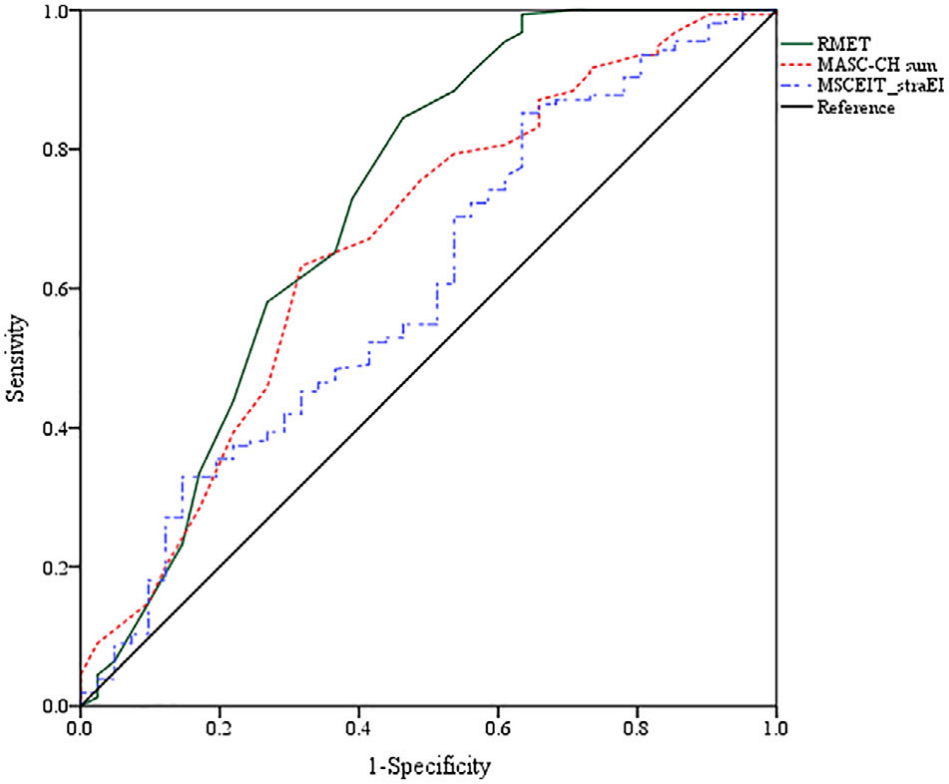
Receiver operating characteristic (ROC) curve of the MASC‐CH in comparison to other measures (MASC‐CH “Movie of Assessment of Social Cognition‐Chinese version,” RMET “Reading the Mind in the Eyes” task, MSCEIT, and Mayer–Salovey– Caruso Emotional Intelligence Test in MD compared to the HC.

To investigate the discriminatory power of the MASC‐CH in between patients with MDD and BD compared to the HC group, the results showed the area under the ROC was .67 for MASC‐CH (*p* = .001), .52 for the MSCEIT_expEI *(p* > .05), .60 for the MSCEIT_straEI (*p* = .04), and .72 for RMET total score (*p* < .0005). An AUC of .50 indicates a possible chance level of diagnostic test, and an AUC with 1.0 indicates the perfect diagnostic test (McNeil & Hanley, 1984). Our results indicated that the RMET, MASC‐CH, and MSCEIT_straEI would be useful to distinguish between patients with mood disorders and HC (Figure 1).

### 3.3 Mental states and error patterns between MDD and BD

**FIGURE 2:**
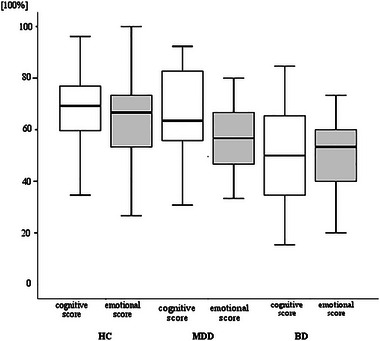
MASC‐CH “emotional mental state” and “cognitive mental state” mentalizing scores (%) from BD, MDD, and HC.

Overall, both patient groups had poor performance on the SC tasks than the HC (Table 2). However, no significant differences were shown between the MDD and HC on subdimensions of each SC task, while the BD had significantly greater impairment on all SC tasks. For the MASC‐CH mental states, the significant influence of both “emotional” and “cognitive” mental states were found as diagnostic difference between the BD and HC, whereas the “cognitive” mental state could be the influent diagnosis between the BD and MDD (Table 2, Figure 2). For the error pattern analysis, the BD and MDD displayed no significant difference on “overmentalizing” error pattern compared to the HC. The BD appeared to have more errors of “undermentalizing” than the HC, and more mistakes of “undermentalizing” and “absent mentalizing” than the MDD. For the MSCEIT, the BD group had significantly lower scores on the MSCEIT total score and MSCEIT‐StraEI score than the HC. For the RMET, the BD significantly displayed the lowest score among the three groups.

**TABLE 2:** Demographic and comparisons of social cognition among groups.

Task	HC (*N* = 156)	BD (*N* = 21)		MDD (*N* = 20)	Statistics χ^2^/*F*(*p*)	Post hoc^a^
Age	40.39 (15.18)	35.57 (10.54)	35.85 (12.18)		1.70 (.19)	–
Gender (male/female)	77/79	7/14	6/14		4.12 (.13)	–
Educational year	15.21 (2.34)	13.40 (3.30)	14.84 (2.57)		**4.73 (.01)**	HC > BD^**^
Onset age	–	22.19 (6.31)	28.00 (10.77)		3.47 (.07)	–
YMRS	–	9.19 (2.66)	–		–	–
HDRS	–	12.00 (4.23)	12.50 (3.42)		0.16 (.69)	–
MASC‐CH sum score	26.88 (5.88)	20.71 (7.93)	25.25 (5.98)		**9.54 (<.0005)**	HC > BD^**^
MASC‐CH mental states						
*“Emotional” mental states*	9.28 (2.26)	7.48 (2.70)	8.35 (2.35)		**6.64 (.002)**	HC > BD^**^
*“Cognitive” mental states*	17.60 (4.18)	13.24 (5.82)	16.90 (4.87)		**8.94 (<.0005)**	HC > BD^***^ MDD > BD^*^
MASC‐CH error responses						
*“overmentalizing”*	5.46 (2.72)	6.48 (2.58)	6.10 (2.63)		1.67 (.19)	–
*“undermentalizing”*	4.87 (2.98)	7.10 (4.06)	5.30 (2.62)		**4.86 (.009)**	HC < BD^**^
Absent mentalizing	3.79 (2.49)	6.71 (4.31)	4.35 (3.59)		**9.76 (<.0005)**	HC < BD^**^ MDD < BD^*^
MSCEIT						
Total score	93.26 (13.13)	85.57 (16.00)	93.69 (9.49)		**3.26 (.04)**	HC > BD^*^
*Experiencing EI*	103.96 (17.74)	96.86 (16.05)	105.29 (10.01)		1.77 (.17)	–
*Strategic EI*	86.49 (9.59)	80.00 (13.21)	85.23 (8.19)		**3.78 (.025)**	HC > BD^*^
RMET	24.73 (3.56)	16.10 (7.52)	21.35 (8.09)		**33.06 (<.0005)**	HC > BD^***^ HC > MDD ^*^ MDD > BD^**^

MDD: major depressive disorder; BD: bipolar disorders; HC: healthy controls. MASC‐CH: Movie of Assessment of Social Cognition—Chinese version; MSCEIT: Mayer–Salovey–Caruso Emotional Intelligence Test; REMT: Reading Eyes in the Mind Test.

* *p* < .05.

** *p* < .01.

*** *p* < .0001.

^a^ Adjusted by Scheffé Test for post hoc.

**FIGURE 3:**
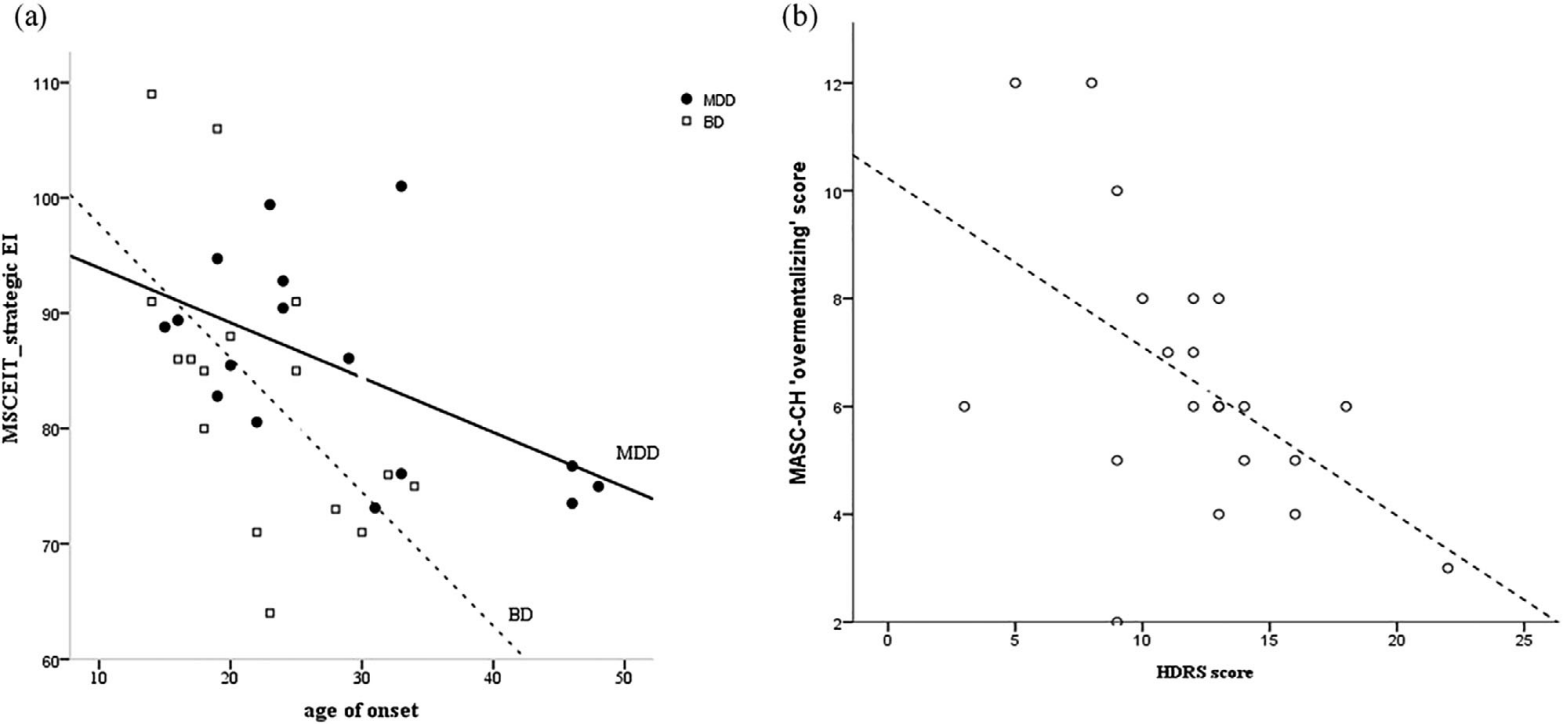
Correlation between clinical characteristics and SC performance in the MDD and BD. (a) Negative association between age onset and MSCEIT_straEI score; (b) negative association between HDRS score and MASC‐CH “overmentalizing” error patterns in the BD.

To explore the correlation between clinical characteristics with SC in MDD and BD, the results revealed a significantly negative association between onset age and MSCEIT total score and MSCEIT_straEI score in BD (*r* = –.59, *p* = .01; *r* = –.60, *p* = .01 respectively), while the negative association in the MDD was only with MSCEIT_straEI (*r* = –.57, *p* = .02). Regarding to the depression severity, the negative association between HDRS and “overmentalizing” error pattern was found in the BD (*r* = –.51, *p* = .01) (Figure 3b), not in the MDD.

## 4 DISCUSSION

### 4.1 Validation Chinese version of dynamic assessment for social cognition

To the best of our knowledge, this is the first study to validate the Chinese version of the MASC according to the original paper (Dziobek et al., 2006). The MASC‐CH was administrated to participants with mood disorders as well as to healthy controls, along with other reference instruments to assess SC. The results showed that the MASC‐CH had acceptable reliability with psychometric characteristics that were similar to the original version (DeVellis, 1991; Dziobek et al., 2006). The MASC‐CH was able to discriminate between the HCs and individuals with mood disorders. Moreover, the BD patients appeared to display greater impairment in SC, as compared to MDD. The BD patients recorded more errors regarding cognitive mental states, as compared to the HC and MDD groups.

In addition, regarding the cultural differences, four items negatively correlated to the MASC and excluded for the analyses are as follows: calling friends on the phone (item 4), feelings of falling in love with others (items 12 and 13), and inappropriate jokes at the dinner table (item 37). The negative correlations could be that these social situations are uncommon in Chinese culture, and the modified MASC‐CH after excluding four items would be more suitable for a Chinese population.

### 4.2 Mentalizing behavioral comparisons between MDD and BD

For the investigation of SC between MDD and BD, we found that the BD patients had greater impairment in SC than the MDD patients, as compared to HCs. The BD group had more errors with undermentalizing patterns and low scores in both emotional and cognitive mental states, implying their difficulties in mental decoding. This finding may reflect the idea that BD patients need more information to identify other people's emotions (Schaefer et al., 2010). In addition, the relatively lower scores on the cognitive and emotional mental scores in BD may indicate that emotional SC is similar to a state marker while the cognitive SC is similar to a trait‐marker. This is consistent with a previous study in remitted BD (Montag et al., 2010).

Previous studies have suggested that the impairment of SC could be accompanied by a severe increase in the symptoms of the BD patients; during the remission state, social‐cognitive ability could be a residual symptom (Judd et al., 2005; Kuo et al., 2021). Moreover, the BD group had lower MASC‐CH cognitive mental state scores than the HCs, while the MDD group showed no difference. This may indicate a possible effect of mood severity in the emotional SC, which is similar to neuropsychological functioning, in that both impair state‐like markers depending on the mood episodes of BD patients (Huang et al., 2020) .

In the MSCEIT test, the EI ability was relatively preserved, and the social cognition ability of BD patients was not completely affected. This result was consistent with the hypothesis that SC could be a multifaceted psychosocial structure (Green et al., 2008), and the impairment of complex advanced SC affected emotional processing and social functioning (Cusi et al., 2012). In a meta‐analysis of 29 studies, the ToM impairment in BD was repeatedly demonstrated. People with BD easily interpreted neutral stimuli as positive; in other words, their sensitivity to rewards may be significantly higher than that of healthy people; the BD patients tended to display impulsivity in their decision making and have difficulties in maintaining interpersonal relationships and occupational functions (Gillissie et al., 2022).

Similarly, Samamé et al. (2015) reported that the patients in symptomatic status showed significant impairments in emotion recognition and social cognition, as compared to the healthy participants. This supported our speculation that impairment of social cognition reduced social functioning in BD, which is also a risk factor for disease recurrence (Gillissie et al., 2022). In our findings, BD patients showed more SC impairment, including the areas of perception, comprehension, and reasoning about the intentions and thoughts of others. A meta‐analysis reported that in addition to the disability of ToM in the symptomatic stage, euthymic BD still have partial ToM disability (Liu et al., 2021; Montag et al., 2010); however, other studies contradicted these findings (Lee et al., 2013; Purcell et al., 2013). This inconsistency could be as result of the multifaceted and complex psychological ability of SC (Achim et al., 2013) and the heterogeneity of BD. A correlation between psychopathology and SC was present in (Vlad et al., 2018), indicating that SC deficiencies could be residual symptoms in a remission state (Lahera et al., 2012; Montag et al., 2010; Samamé, 2013) and an endo‐phenotype in BD (Liu et al., 2021).

There was no difference between MDD and BD in some dimensions of SC, implying a similar psychopathology between two types of mood disorders. van Neerven et al. (2021) reported that patients with MDD had significantly lower MASC scores than the HCs and showed a tendency for undermentalizing error patterns (van Neerven et al., 2021), although this was not found in the current study due to MDD patients in our study were with mild depression. A decreased high‐order SC function found in MDD with moderate‐to‐severe first‐episode depression (HamD‐17 *M* = 22.3, SD = 3.8) was suggested (Ladegaard et al., 2014), and it was documented in severe MDD (Lee et al., 2005). The cognitive impairment in MDD fluctuated with symptom severity was suggested (Bora & Berk, 2016). Weightman et al. (2014) have suggested that SC impairment in MDD appeared to vary and be reversible with treatment (Weightman et al., 2014), which was in agreement with previous conclusions that SC impairment in MDD could be a state‐like symptom (Berecz et al., 2016).

The negative association with onset age and different SC tasks revealed different effect in between the MDD and BD. In both patient groups, a negative association between MSCEIT_straEI scores and age onset was found, indicating the effect of age onset on strategic emotional management ability in patients with mood disorders. Moreover, the impact of depression severity on MASC‐CH overmentalizing error was only noticed in the BD, indicating a greater effect of depressive symptoms on higher functions (Dodd et al., 2019; Judd et al., 2005; Tabak et al., 2015). The association between depressive symptoms and MASC‐CH mistake of “overmentalizing” in our study confirmed the greater impact of depression on SC.

### 4.3 Limitations and contributions

There were some limitations in our study. First, the sample size of our patient groups was small, although we have justified using Scheffee post hoc for the multiple comparisons among unequal groups (McHugh, 2011); a larger sample is needed to be able to generalize the results. The modified MASC‐CH was found with acceptable validity and reliability after excluding four items, implying a cultural component should be considered, which has been reported in emotional recognition (Leffers & Coley, 2021; Matsumoto, 1992). Further studies conducted with culturally familiar aspects and social situations may be needed for further confirmation of the MASC‐CH as a reliable instrument for measuring dynamic SC. Moreover, the MASC‐CH was found as a useful instrument for discriminability between BD and HC, not between MDD and HC. Although the MDD and BD patients were with mild severity, the effects of mania may play an important role in SC for BD. The negative correlation between depression severity and SC was only in the BD group, not in the MDD, so the possible impact of depression symptoms could be different between MDD and BD. Studies in neuroimaging have indicated that MDD had enhanced top‐down control between the prefrontal cortex and the amygdala, particularly in positive facial expressions, while BD depression had reduced prefrontal control over amygdala reactivity (Almeida et al., 2009). Further longitudinal studies should be conducted to investigate the trajectory of SC impairment in MDD and BD, and whether SC impairment is part of the psychopathological characteristics in mood disorders.

## 5 CONCLUSIONS

In summary, the modified Chinese version of the MASC (MASC‐CH) was validated and reliable. In addition, the BD group showed more SC impairment than the MDD group, as compared to the HC group. The results indicated a deficit in cognitive mental state attribution in BD that could be a result of their “undermentalizing” and deficiencies in the higher strategic and reasoning abilities in emotional intelligence. Such deficits are likely to interact with depression psychopathology in BD, but not in MDD. The etiology between MDD and BD warrants further studies on the other aspects of SC and the extent of impairments in different stages of BD and MDD.

## AUTHOR CONTRIBUTIONS

YHC designed the study, and wrote the first draft with CLY. CLY, CCH, and TYW managed the patient recruitment with HYL. CLY performed the data analyses and interpretation under supervision by YHC. ID and HYL gave feedback on the analysis and the manuscript. All authors read and approved the final manuscript.

## CONFLICT OF INTEREST STATEMENT

All authors have reported no conflict of interest.

### PEER REVIEW

The peer review history for this article is available at https://publons.com/publon/10.1002/brb3.3365.

## Data Availability

Data are available on request from the authors.
